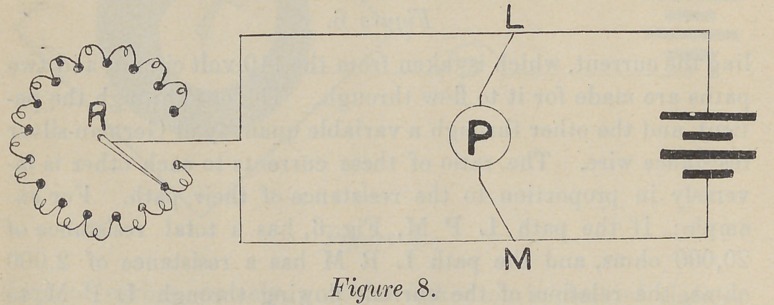# Relative Efficiency of Various Current-Controllers for Cataphoresis

**Published:** 1897-02

**Authors:** W. A. Price


### Relative Efficiency of Various Current-Controllers for Cataphoresis.

BY W. A. PRICE, D. D. S.

Read before the Ohio State Dental Society, at Columbus, O., December 2, 1896.

It is my purpose, in this paper, to confine the discussionto the various principles used, and not to mention individual instruments.

I must express my obligations to Professors Miller, Langley and Carter, all of Case School of Applied Science, for their excellent assistance in experiments and tests and for the use of very excellent scientific apparatus, without which I could have done but little.

The function of a controller is to furnish an electric-current absolutely at the will of the operator. There are seven distinct varieties of instruments on the market for this purpose. Let us note the distinctive characteristics of each.

First, the water rheostat, Fig. 1, which is placed in series

*Figure* 1.

with the battery and patient. One pole of the current is placed in the bottom of a jar of water, J; and the other attached to a sliding-post, R. The current passes through the patient, the water, and the batteries in series, and is controlled by varying the distance between the end of "R" and the bottom of the jar, the amount of current being controlled by the amount of water.

The next, Fig. 2, is constructed on the same principle, except

*Figure* 2.

that the current goes through fine German-silver wire, instead of water, and the amount of current is controlled and varied by the amount of wire put in the circuit. As in Fig. 1, the current passes through the patient, the rheostat and the batteries in series.

Fig. 3 is identical in principle, but instead of passing the

*Figure* 3.

current through fine wire, it is passed through green carbon of relatively a very high resistance. The piece of carbon has little screws inserted, very close together, and the resistance is increased by moving the contact-lever further from the end, to which is attached the other pole of the current.

Fig. 4 is very similar to figure 3, except that it is another

*Figure* 4.

form of carbon, namely, graphite baked on a piece of slate or glass. The current is controlled by the position of the lever, which has a metallic-brush-contact with the graphite. As in all the preceding forms, the current goes through the patient, rheostat and the batteries in series.

In the next, Fig. 5, the current is taken from the 110-volt

*Figure* 5.

circuit and passed through sufficient resistance, usually graphite or powdered carbon, to cut it down to a sufficiently-low potential. Necessarily, a much larger resistance is used.

In Fig. 6 we have an entirely different principle for control-

*Figure* 6.

ling the current, which is taken from the 110-volt circuit, and two paths are made for it to flow through. The one through the patient, and the other through a variable quantity of German-silver resistance wire. The ratio of these currents to each other is inversely in proportion to the resistance of their path. For example: If the path L P M, Fig. 6, has a total resistance of 20,000 ohms, and the path L R M has a resistance of 2,000 ohms, the relation of the current flowing through L P M, to the total current flowing, is as 2,000 is to 20,000 plus 2,000, or one-eleventh, ten-elevenths flowing through L R M.

By varying the relation of the resistance in these two paths, the current in both is varied; so since the resistance of the patient is fixed, the current in that path, viz., L P M, is varied by changing the resistance in the path L R M. By increasing the resistance of L R M the total amount of current flowing through the two paths is diminished in proportion to the total increase of resistance of the combined paths. The next, Fig. 7,

*Figure* 7.

is similar to the last, except that it has a three-way shunt. Two of them are constant, viz., L P M, in which the patient is the resistance, and L S M, which is a definite amount of German-silver wire. The third, L R M, has a variable amount of resistance wire, and the relative amount of current flowing through these paths is varied according to the relation of their resistance, always in inverse proportion, and is varied by changing the amount of resistance in L R M. In the last, Fig. 8, the prin-

*Figure* 8.

ciple is identical with Fig. 6, the difference being only in the source of current. In this case it is batteries instead of the 110-volt circuit.

Just here let us review some of the laws of electricity: 1. The amount of current flowing depends on two things—the electromotive force, or potential, or voltage, and the amount of resistance in the path.

The unit of electric-pressure is a volt; the unit of current-strength is the ampere, and is the strength of current necessary to decompose .09326 milligrams of water in one second. A milliampere is the one-thousandth part of an ampere. The unit of resistance is an ohm, and is the amount of resistance through which 1-volt pressure will produce one ampere of current. This is simply one way of expressing Ohm's law, which is: That the electric pressure, divided by the resistance, is equal to the current. The electric-pressure is expressed as the voltage—the resistance as ohms, and the amount of current flowing, as amperes. The volts over the ohms equal the amperes; or, the volts over the amperes equal the ohms. Doubtless you are all very familiar with these terms, but I review them for fear some one is not, and I shall use them hereafter entirely to express these quantities.

Let us next consider the conditions in the patient as the current passes in through the medicine and tooth and out through the body at some more or less distant point. The amount of work done in a given case depends upon the amount of current flowing, and the amount of current is fixed by the pain-limit. Two things are practically definite in every individual cavity, viz., the resistance through the patient and the pain-limit. The former is, relatively, a different quantity in different patients, and in different cavities of the same patient, and as a more or less remote point on the body is used from which to take the current. The average resistance through the patient, for about twenty-five cases, was about 25,000 ohms, varying all the way from 10,000 to 78,000 ohms, and in some cases even higher. The difference of resistance from the hand to the tooth, and the cheek to the tooth, is from 3,000 to 5,000 ohms. It is almost incredible, the amount the resistance can be varied by the condition of the cavity. For example: In a given case the resistance through the patient from the cavity, which was barely moist, to the hand, was 47,700 ohms. A 40-percent solution of cocaine, in water, placed in the cavity, reduced the total resistance to 28,500 ohms, and placing the pad on the cheek instead of the hand, reduced it to 23,800 ohms. These measurements are approximately accurate, for they were made by standard instruments, most of them from the source named above. This is only a fair sample of many cases I have the figures for here at this time.

Measurements of liquid resistances, such as the body, can not be made with a Wheatstone bridge, as can solid substances, owing to a secondary induced current set up by the electrolysis of the fluid. They can best be calculated by Ohm's law. For example: An accurate volt-meter reading, in tenths, indicates a difference of potential across the patient of 5.7 volts, and a milliammeter reading in hundredths of thousands of amperes, indicated twenty-hundred-thousandths, then the resistance of a patient would equal 5.7 over twenty-hundred-thousandths, or 28,500 ohms. Ordinary commercial milliammeters that I have tried to use, and compared with the standard instruments, would not register with any degree of accuracy through the first few degrees of the scale, the only part needed—nor would volt-meters. The milliammeter used for most of these measurements could be adjusted to read in any fraction of an ampere from one-thousandth to one-millionth. The average resistance from the hand to the tongue, with small electrodes, is about 9,000 ohms, varying from 7,000 to 12,000, and from the cheek to the tongue about 5,000 ohms, varying from 3,000 to 7,000. It will be seen at a glance that by far the greater part of the resistance of the patient is in the tooth, varying all the way from 1,000 to 70,000 ohms—an average of probably near 20,000 ohms.

Measuring cavities at different stages during the excavating, with as nearly as possible the same conditions, show a gradual decrease of the total resistance—in many cases many thousands of ohms.

Time will not permit of many figures on the relative resistance of dentine taken from different parts of the same tooth and from different teeth, and the variation of resistance of the same section of dentine, according as it is saturated with different solutions. For example, a longitudinal section of fresh dentine almost dry on the surface, and five millimeters in thickness, had a resistance of 30,000 ohms, after dehydrating and saturating with a forty percent solution of cocaine the resistance was reduced to 4,500 ohms, and on again dehydrating and saturating with a sodium chloride solution (common salt), the resistance was reduced to 3,070 ohms. The bearing of this on the process of cataphoresis will develop later.

Is the pain limit variable for a given cavity? *Yes*, though it is normally almost constant, except when medicated, in a case where there is no inflammation of the dentine or pulp, and where *the patient's general nerve tone is constant.*

Is there any *physical* difference in a *constant* and perfectly regular electric current of a given strength, however it may be produced? This is probably the most universal question in the minds of the members of our profession at this stage of the advancement of cataphoresis. No, there is not, according to the opinions of all electrical authorities I have been able to find, provided the current be perfectly *constant* and of a given strength. This will seem a contradiction to the experience of many of you who have used currents derived from different sources, as it did to myself. We will try it in a few minutes.

The next may seem a greater contradiction to some of your experiences. Is there any difference in the physiological effect of a perfectly constant electric current, however it may be produced, provided the conditions remain the same and it is without variation? Answer, *No.* I mean by that that a current of one-ten-thousandths of an ampere produced by passing a current of four volts through 40,000 ohms resistance, say 25,000 ohms resistance in the patient, and 15,000 ohms resistance placed in the circuit, will cause identically the same amount of pain as a current produced by passing a current of eighteen volts through 180,000 ohms resistance, or a current produced by passing seventy-two volts through 720,000 ohms resistance, or a current produced by passing one hundred and eight volts through 1,080,000 ohms resistance, provided it is turned on in such a way as not to produce shock.

I have done this frequently in my practice, as follows: The reflecting galvanometer was set to read 240 points to the milliampere. A current with a difference of potential between the poles of the batteries of six volts was allowed to pass through the patient and some resistance. The resistance was cut out gradually until the patient felt a definite sensation of pain. At this point the milliammeter indicated 142 points, or $\frac{142}{240}$ of a milliampere. This current was cut off carefully. Next, a current with a difference of potential between the poles of the battery of eighteen volts was allowed to pass through a much higher resistance, and the patient was instructed to give a signal when the same definite sensation was felt by reducing the resistance which he did when the needle stood at just 142 points. This current was cut off and one of twenty-one volts through a higher resistance passed and the patient instructed to signal when the same definite sensation was produced by cutting out the resistance very gradually. He did so and the needle stood at just precisely 142. This was stopped and forty-eight volts different of potential passed through a higher resistance and the patient's signal was given when the needle was at just 142 points. Then a current of one hundred and eight volts was adjusted in the same manner, and the signal came when the needle was at just 142 points. I have done this frequently with myself and with a great many of my patients, and have alway gotten the same results, though some patients could not determine a definite sensation as accurately as others. I have sufficient apparatus here and will assist any of you to make the experiment upon yourself at the close of the session. If you try the experiment be sure to fasten the electrodes firmly in their respective positions, and do not disturb them a particle throughout all the tests.

I think I can demonstrate it so you all can see with the reflex produced on this frog's leg. You see that by turning the current on very gradually, so as not to produce the least sudden stimulation, there is actually no difference in the angle of contraction produced by these different currents. This means, if all the conditions necessary have been covered, that one apparatus that will perfectly control the current is just as good as any other. But have all the conditions necessary been covered? *No!* I have here like resistances of the various substances used for that purpose, each with a total resistance of 45,800 ohms, and if the statement is true that a current of definite strength is physically the same, it matters not how it is obtained, then the same electro-motive force passed through these different materials of equal resistance should give the same physiological effect. You put this electrode on your tongue and you can not distinguish any difference in the sensation. The sensation of feeling seems to be identical with the indications presented in the reflex of this frog's leg. You can all see it and you can try the experiment on your tongues afterwards. These resistances are German silver wire, water and graphite, and are connected alternately on this switchboard for convenience, and you can not detect any perceptible difference in the reflex of the frog's leg or the sensation on your tongue by passing the current through one or the other of these substances. This answers the question whether any one substance used as the medium of resistance produces any more or less pain than any other substance, *all the conditions remaining the same.* It certainly does not, although we hear so many assertions to the contrary.

Let us repeat what was asserted before, that a perfectly regular and *constant* current of definite strength will produce the same physiological effect under the same conditions, no matter how produced. If you apply two electrodes to your tongue it is a broken circuit, not a constant one. What difference does it make whether a current is constant or not? I wish you could each try this next experiment upon your tongues, although this frog's leg reflex will demonstrate it. We will use as nearly as possible the same current strength in every case as indicated by this galvanometer. In the first case we will use a voltage of one and one-half volts, and pass it through 3,840 ohms resistance.

In the next 18 volts through 45,800 ohms, and in the next 110 volts through 282 050 ohms—in each we will get a current-strength of thirty-nine one-hundredths of a milliammeter. With the first you see but a slight, though definite, reflex of the frog's leg; with the next, though identically the same amount of current, there is a decidedly greater reflex; and with the last a still greater increase of the strength of the reflex. What is the explanation? Suppose you have three pumps so regulated with governors that they will keep just thirty-nine barrels of water circulating, per minute, through each of three separate, complete circuit systems of pipes. The first system has just enough miles of pipe so that the pressure that accumulates at the pump to force that amount of water around the circuit, per minute, is 1½ pounds to the square inch. In the second system there are enough miles of pipe so that the pressure at the pump, as it sends out the water, is 18 pounds per square inch. In the third there are just enough miles of pipe to produce a pressure of 110 pounds per square inch at the outgoing side of the pump, in order to force the thirty-nine barrels around through the circuit, per minute. Now suppose, in the first, a check of some kind is put anywhere in the circuit so no water can get past, then the pressure will rise all the way along in the pipe, as far as the check, to 1½ pounds to the square inch, that being the limit of the pump's pressure. Suppose a check is put in anywhere in system No. 2, and the pressure will rise all along that system, to the check, to 18 pounds per square inch—and so with the third system, the pressure all the way from the pump to the check would rise to 110 pounds per square inch. Let us suppose one pound to be the pressure required to force thirty-nine barrels of water through one mile of pipe, per minute. Now, suppose those checks were to be suddenly removed, what would be the strength of current for the first instant? In the first case it would be at the rate of 1½ times thirty-nine barrels per minute, or 58½ barrels. In the second, at the rate of 702 barrels per minute, and in the third at the rate of 4,290 barrels per minute. This rate would last for only an instant, and would immediately decrease until it reached its normal rate. The time this increased rate would last could hardly be calculated, but it exists, and we have all observed it at the hydrant. This is precisely what occurs in the circuit of an electric current when it is broken. This produces what is called the throw of a needle of a current-meter, some of which are constructed to not register this first impulse. The difference in the effect according as the plug is near to or far from the pump, will come up later. Although there is such a difference in the reflex produced by these two currents, by a sudden make or break, you will see there is no difference in the amount of work they will accomplish, for we will now connect them with these tubes of iodide of pot., and the amount of electrolysis is identical. If I turn these various currents on slowly enough, there is no perceptible difference in the reflex produced on this frog's leg, or the pain produced on the tongue or in a tooth. It is not necessary to break a circuit to get this effect, for it takes place to a greater or less extent with every variation of potential, or of the total resistance of the circuit.

There are certain facts accompanying the process of cataphoresis which materially determine the requirements of a satisfactory controller. First, the current-strength can not remain constant, it must be continually increased as the pain-limit will admit, owing to the anæsthetizing of the tooth. This is accomplished in two ways, either by increasing the voltage or by diminishing the resistance of the circuit. The former causes pain if done too rapidly or in too large quantities, and that in some cases as low as one-twentieth of a volt; the latter produces pain if diminished too rapidly or in too large quantities. Even if there is 50,000 ohms resistance in the circuit, and the total is reduced by 100 ohms, it will frequently produce pain. Another fact attending this operation is, that the total resistance of the circuit can not be kept constant. Any movement of the electrodes, especially the one in the tooth, though ever so slight, may vary the total resistance of the circuit to almost any amount. No operator should ever attempt to hold the electrode in his hand, for it is impossible to prevent causing some variation in resistance of the circuit at that point. It is not necessary. Flexible, fine gold or platinum wire (which by the way dental depots would do well to furnish; about $\frac{4}{1000}$ of an inch in diameter), packed into the cavity with cotton carrying the medicament will be infinitely more satisfactory than any of the excellent clamps for that purpose. In over 300 cases I have not required to hold it in a dozen. A special device can be made in a couple of minutes for any special case. Another source of variation of the total resistance is from the cavity, or the pad drying out, and when moistened produces shock by a sudden increase of current, due to lowering the total resistance. It is absolutely impossible for us to keep a constant current. We would not if we could, and we could not if we would.

Since the current can not be kept constant, we should keep the total resistance in the circuit as low as possible. If using batteries with resistance in series, do not use any more cells than will do the work. For while, if the current were to remain absolutely constant, there would be no difference in the pain produced, since that is clearly impossible, and there must and will be variations; for the many reasons given there will be more intense shocks caused by the same variation. If you get an apparatus in which the resistance is in series with the patient and batteries, be sure it is wired so you can turn on one cell at a time. I have had cases where 50,000 ohms resistance in series with one cell and the patient produced unbearable pain, and the pain was not all relieved until it was increased to 108,000 ohms. What would have been the effect if I could not use less than twelve or fifteen or twenty cells in series, or had a total resistance of not more than 1,000 or 5,000 as some instruments have? An apparatus to be sufficient for all these extremely-sensitive cases should, if resistance is put in series, have a total variation of not less than 100,000 ohms. Probably not over 10 percent of cases will require over 70,000, nor more than 25 percent over 40,000.

Let us next consider the other principle for controlling the current, viz., by means of a shunt. Suppose the resistance of L P M to be 25,000 ohms, which is, of course, the resistance of the patient, and the difference of potential between L and M is 110 volts, what must be the resistance of L R M to cause one milliampere of current to flow through L P M? It will require some introduced resistance at L or M to limit the amount of current within easy control, let us say two lamps of 275 ohms resistance each, and according to the law of inverse proportions, the current flowing through L P M is to the current flowing through L R M as the resistance of L R M is to the resistance o L P M. The resistance of L R M approximately is 160 ohms.

Suppose the resistance of the patient be 40,000 ohms, then the resistance of the other path, L R M, will be approximately 325 ohms. If L P M equal 10,000 ohms, then to give one milliampere, L R M equals approximately 58 ohms. You will see at once that the amount of resistance placed in the shunt L R M is no indication of the amount of current flowing through L P M, unless you know the resistance of L P M. Hence, since the resistance of patients is such an uncertain and widely varying quantity in different cavities, it is impossible to arrange any scale of indication of the actual amount of current any given case is getting. If the resistance of the patient was universally one ohm, or any other definite quantity, or any approximate quantity, then an arbitrary scale could be just as approximately correct. Under the existing positively uncertain conditions they have practically no definite significance. This includes the indicators of so-called volt-selecters. This problem of shunts is very complicated and I will dwell on it at greater length in my closing discussion. (See discussion.)

What is the effect of breaking, or making the current of one path of a two-way shunt? You get the actual true current strength as the first sensation. For example, suppose the resistance of a patient be 20,000 ohms, and one-tenth of a milliampere is flowing through the patient's circuit L P M, then the difference of potential between the two sides of a patient would give the same amount of shock that he would get from a cell giving two volts when there was no resistance in series. If the resistance of a patient were 50,000 ohms, and one-twentieth of a milliampere of current was flowing, then the difference of potential across the patient would give the same shock as ten volts in series and the patient would get the same shock which he would from five cells in series giving two volts each. You see by this method the minimum possible shock is given, and since the variation is inevitable in the current strength, this is a very important item. This is very easily demonstrated by the frog's leg reflex and felt by the tongue.

Now, a word as to the capacity and necessary requirements of any controller to do all cases absolutely painlessly and thoroughly, which is possible in ninty-five percent of cases. The instrument should be able to furnish a difference of potential across the patient of at least twenty volts, for actual measurement of cases show that some will finally stand that amount of current. With an instrument taking the current from batteries with the resistance in series—this simply means that there be enough cells to produce that voltage—but if a shunt from a one hundred and ten volt circuit it should have variable resistance in the shunt in proportion to the permanent resistance put in the circuit. If a shunt from batteries, the batteries should have a total voltage of thirty volts, and the cells should be arranged with a cell selecter and high variable resistance. This is one objection to a shunt on cells, that a greater variable resistance is necessary owing to the lower potential unless a very large number are used. It is not a practical objection, however, since the alternative is very practicable. It is clearly evident that a shunt is the most satisfactory means of controlling the current by far, from the experiments we have made, no matter from whence the source of the current, economy not considered.

The economy of the cells is an important factor, and since a shunt of low resistance is simply a short circuit, the cells will not last so long as if less are used and a higher resistance in the shunt. What are the shortcomings and points of superiority of these different methods we have considered?

First. The water rheostat. If platinum electrodes are used in distilled water, and the total resistance is high enough, not less than 100,000 ohms, which means a column of about twelve inches, according to the size of the electrode, and the mechanical working of the machine is such that a perfectly smooth and easy control is had of the current, it is just as good as any, using identically this same principle. The majority of this variety have not one-quarter enough variation of resistance. Four inches of lake or river water would have about 5,000 ohms resistance, of salt water less than 1,000 ohms resistance. Draw your own conclusions.

The German silver resistance in the circuit in series is efficient accordingly as it is finely divided into steps, or is sufficient in quantity, there should be 100,000 ohms with steps of not more than one hundred ohms each for an ideal machine. It is probably as cleanly and durable as any. It is a fact, however, that the metallic-contact connections, whether the points of a switch, or a brush, on a coil, are not constant, and I have not seen an instrument in which they were a part that would not show a fluctuation of current caused by them. Not enough to be an excluding objection if kept clean.

The objections to the metallic-contact points in the green carbon stick are the above, and that there is not possible a great enough total variation of resistance, and the contact points can not be closely enough together to prevent shock unless a very large board be used.

A system in which, as in Fig. 5, there is a very high resistance in the series in the one hundred and ten volt circuit is not consistent, nor could its results be satisfactory. A difficulty with any form of lever to be moved by the hand over any carbon or graphite surface, is that the distance it can be moved without producing shock is too small unless the instrument be larger than are generally used. Powdered carbon is extremely variable in its resistance. Where a three-way shunt is provided, more variation is required in the variable shunt if its resistance is low. It is considered a guard against shock from variation of potential.

We have not referred to the automatic devices for increasing the potential, or diminishing the resistance, all are good and are practical, many are very excellent, but need constant attention. This feature has not been appreciated by manufacturers in general. What are the logical conclusions to draw from the foregoing experiments?

First: A method in which the principle of the device used for controlling the current is a shunt is decidedly preferable to one where the resistance is put in series in the circuit.

Second. Keep the total resistance of the circuit as low as possible.

Third. Remember that the sensation produced by suddenly breaking the current is very similar to that of making it suddenly, not identical, however.

Fourth. If the one hundred and ten volt current is without variation of potential it is of almost equal efficiency with batteries as a source of current, if a shunt system is used. Not so if in series. It is not absolutely constant, however, in all cities, nor in all branches where the mains themselves are fairly constant. In many cities the direction of the current is liable to be changed. Both of these are important items, the former would produce shock and the latter absolute failure, and, I believe very frequently does.

Fifth. Always keep just below the pain limit.

Sixth. The fluctuations sometimes felt by the patient are not always produced by a change of current; they are sometimes physiological in origin.

Seventh. After testing a dozen different varieties of instruments, and recording over three hundred cases in which absolute failures were about two percent, partial failures about eight percent, and in which over thirty pulps were drilled out absolutely painlessly.

I conclude that some principles are inconsistent and the instruments using them failures, some all right in some cases, some excellent considering, but as yet none are just what the profession should demand and will some day get.

## Figures and Tables

**Figure 1. f1:**
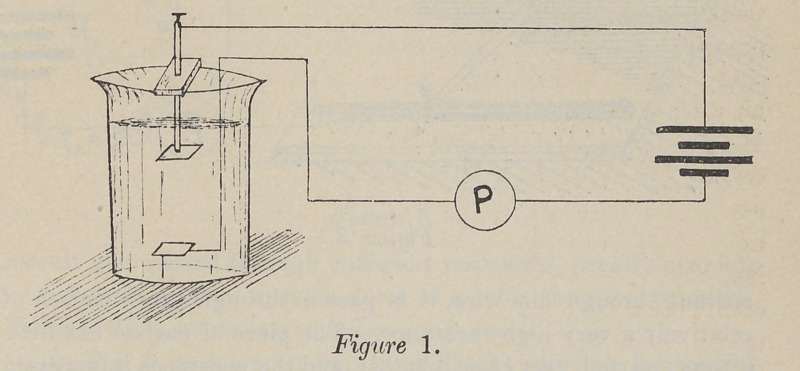


**Figure 2. f2:**
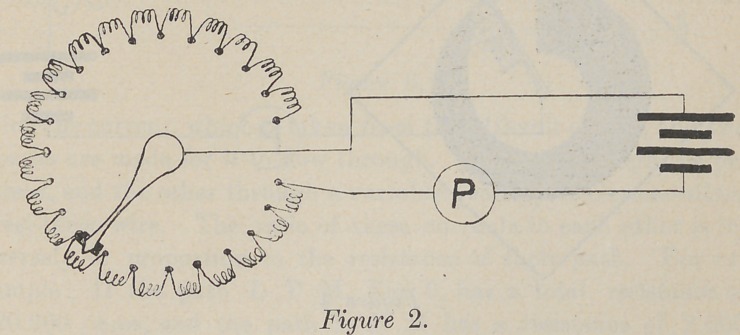


**Figure 3. f3:**
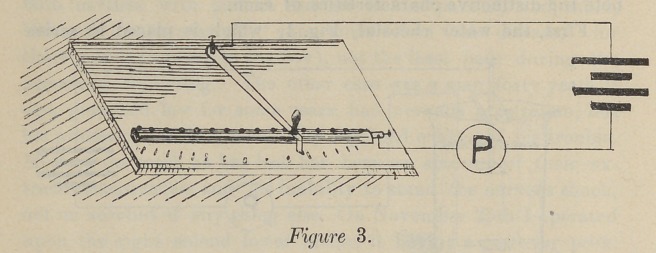


**Figure 4. f4:**
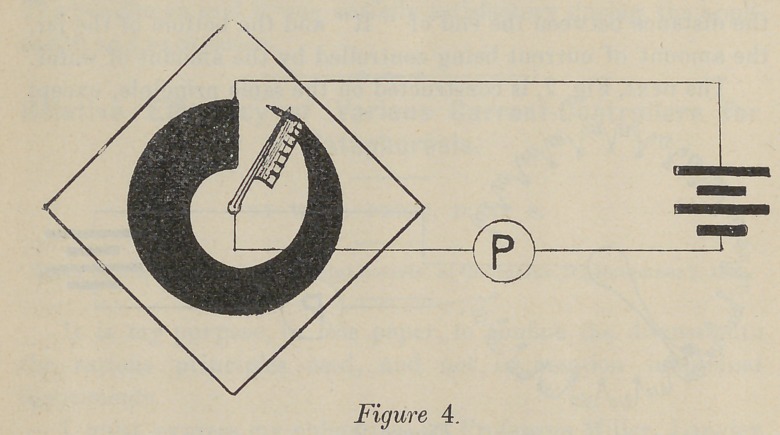


**Figure 5. f5:**
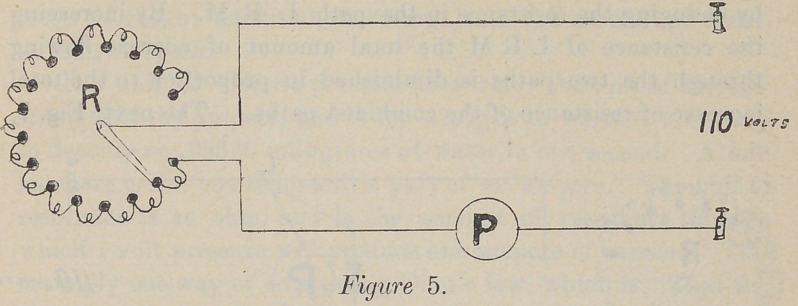


**Figure 6. f6:**
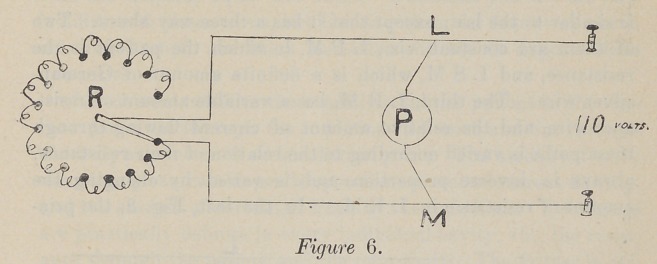


**Figure 7. f7:**
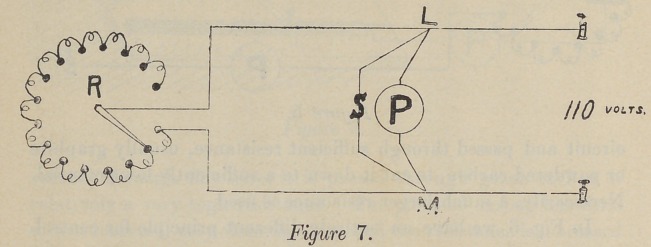


**Figure 8. f8:**